# Influencing Factors of Pregnant Women’s Willingness to Receive Influenza Vaccination in China

**DOI:** 10.3390/vaccines13121194

**Published:** 2025-11-26

**Authors:** Yi Tao, Nan Shan, Yan Wang, Minghong Sun, Lijuan Zhang, Lihong Yang, Lei Chen, Hongbo Qi, Junlong Li

**Affiliations:** 1 Phase I Clinical Trial Ward, The First Affiliated Hospital of Chongqing Medical University, Chongqing 400016, China; taoyi@hospital.cqmu.edu.cn (Y.T.); 202011@hospital.cqmu.edu.cn (Y.W.); 204693@hospital.cqmu.edu.cn (M.S.); 204817@hospital.cqmu.edu.cn (L.Z.); 204581@hospital.cqmu.edu.cn (L.Y.); 2 Department of Obstetrics, The First Affiliated Hospital of Chongqing Medical University, Chongqing 400016, China; 204213@hospital.cqmu.edu.cn; 3 Chongqing Key Laboratory of Maternal and Fetal Medicine, Chongqing Medical University, Chongqing 400016, China; 4 Chongqing Yuzhong Center for Disease Prevention and Control, Chongqing 400016, China; drury@163.com; 5 Joint International Research Laboratory of Reproduction and Development of Chinese Ministry of Education, Chongqing Medical University, Chongqing 400016, China

**Keywords:** pregnant women, influenza, influenza vaccination, willingness

## Abstract

Background: Seasonal influenza presents a major public health issue with a heavy disease burden on pregnant women, who are at increased risk of severe outcomes, yet vaccination uptake remains low. Objective: Investigate pregnant women’s willingness to receive influenza vaccination, identify key influencing factors, and elucidate the pathways shaping this willingness. Methods: A cross-sectional web-based survey was performed. The questionnaire included their socioeconomic characteristics, pregnant status and histories, knowledge of the influenza vaccine, and attitudes toward the seasonal influenza vaccine. A univariate analysis was initially performed to screen potential variables associated with pregnant women’s willingness to receive influenza vaccination. Subsequently, a multivariate binary logistic regression model was constructed to assess the independent effects of these variables on vaccination willingness. Additionally, a mediation effect model was developed to analyze the mediating pathways of factors influencing vaccination willingness. Results: A total of 1231 pregnant women participated in this survey. Five hundred and seventy-one (46.4%) were willing to receive the seasonal influenza vaccine. Multivariate analysis identified five key factors influencing willingness: higher annual income (CNY ≥ 200,000 vs. ≤100,000: OR = 2.65, 95% CI: 1.02–6.85), history of delivery difficulties (OR = 0.10, 95% CI: 0.02–0.62), knowledge about the influenza vaccine (OR = 1.69, 95% CI: 1.28–2.23), supportive attitudes towards vaccination (OR = 13.35, 95% CI: 8.26–21.55), and willingness to pay over 500 yuan for the vaccine (OR = 2.29, 95% CI: 1.23–4.27). Attitudes towards vaccination were partially mediated by vaccine knowledge (β = 0.03, 16% of total effect), with no mediation effects observed for price or delivery history. Conclusions: This study pinpointed pivotal factors contributing to pregnant women’s hesitancy towards seasonal influenza vaccination. Our findings underscore the urgent need for integrating influenza vaccination programs with comprehensive educational and promotional strategies in the future.

## 1. Introduction

Seasonal influenza infection is a global health concern, circulating worldwide and resulting in a substantial annual burden of morbidity and mortality. Pregnant individuals, along with their newborns, face a notably elevated risk of experiencing serious complications stemming from influenza. When compared to non-pregnant women, expectant mothers are more likely to be hospitalized due to influenza-related complications, with this risk being particularly pronounced among those who have pre-existing health issues [1]. Moreover, contracting influenza while pregnant can lead to unfavorable outcomes for both the mother and the fetus, including premature birth or even fetal demise [2]. Data from the influenza seasons spanning 2010 to 2017 in the United States reveals that pregnant women made up 24–34% of all influenza-related hospitalizations among women in the 15–44 age bracket [1]. The likelihood of being hospitalized because of influenza is four times greater during pregnancy compared to non-pregnant times [3]. This situation underscores the critical and immediate necessity to enhance preventive and control strategies for seasonal influenza among pregnant women, aiming to mitigate the health risks associated with it.

Currently, there is no officially licensed immunogenic influenza vaccine available for infants under six months of age [4]. Against such a backdrop, maternal influenza vaccination emerges as the most promising strategy for preventing influenza infections in infants during their initial months of existence. The act of getting vaccinated against influenza fulfills a dual protective role, offering advantages to both expectant mothers and their newborns. Numerous studies have demonstrated that influenza vaccination acts as a shield against potentially serious health consequences. It has the potential to lower the risk of complications and hospitalizations associated with influenza [5]. Moreover, maternal immunization offers an additional layer of protection for infants under 6 months. Through the process of transplacental antibody transmission, maternal antibodies generated in response to the influenza vaccine are transferred to the fetus, significantly reducing the likelihood of influenza illness in the vulnerable neonatal period [6,7].

Randomized controlled trials (RCTs) have demonstrated that seasonal influenza vaccination administered during pregnancy yields a 56% efficacy (95% CI: 28–73%) in preventing influenza virus infection among infants under 2 months and a 35% efficacy (95% CI: 19–47%) in those under 6 months [7]. Observational studies have revealed that seasonal influenza vaccination during pregnancy exhibits an efficacy range of 41% to 71% in preventing influenza virus infection in infants [7,8]. Additionally, it has shown an efficacy range of 39% to 92% against hospitalization. A synthesis of existing literature reveals no documented cases of adverse pregnancy effects associated with influenza immunization during pregnancy [9]. Since 2012, the guidelines issued by the World Health Organization (WHO) have clearly identified pregnant women as a high-priority demographic for inclusion in national influenza vaccination campaigns [10]. This designation is particularly emphasized for women in the second and third trimesters of pregnancy, as well as postpartum women. Additionally, a vast majority of European nations have implemented policies endorsing influenza vaccination for pregnant women. Data from the Centers for Disease Control and Prevention (CDC) indicate that approximately 57.3% of expectant mothers in the United States received an influenza vaccine during the 2022–2023 flu season. In contrast, China’s situation remains ambiguous, as pregnant women’s perceptions of seasonal influenza vaccines and the root causes of consistently low uptake rates require further exploration [11]. Since 2018, China has designated pregnant women as a priority group for influenza vaccination and recommended voluntary self-funded vaccination; however, due to pharmacopeia restrictions, a lack of data, and low public awareness, the actual vaccination rate remains extremely low, necessitating policy revisions, expanded free coverage, and enhanced public education to improve uptake [12].

In this study, our primary objective was to explore pregnant women’s willingness to receive influenza vaccination and to pinpoint the factors that influence this willingness. Additionally, we delineated the pathways that shape their willingness toward influenza vaccination. We aim to furnish valuable insights that can inform the development of policies for the national immunization program.

## 2. Materials and Methods

### 2.1. Study Design and Population

From 24 February to 1 June 2025, we conducted a cross-sectional study by using a non-probability sampling method through WeChat from the phase I clinical trial ward of the first affiliated hospital of Chongqing Medical University (Chongqing, China). To mitigate the risk of overfitting and enhance the generalizability of the results, the sample size ought to encompass 10–20 observations for each variable. Consequently, this study is projected to necessitate a sample size ranging from 500 to 1000 [13]. Pregnant women were invited to participate in an online survey to assess their willingness toward the influenza vaccination. All participants were explicitly informed that questionnaire responses would remain anonymous, and study participation was entirely voluntary. They were guaranteed that their personal data would be used exclusively for research purposes and that questionnaire answers would not affect their prenatal care. Eligibility criteria required participants to (1) be aged 18 or older; (2) be at least 12 weeks pregnant; (3) reside in mainland China; and (4) complete over 95% of the questionnaire items. Participants who failed to answer 95% of the questions correctly were excluded. To prevent duplicate or fraudulent entries, responses were cross-checked using age and IP address verification.

### 2.2. Measures

Socioeconomics—Characteristics included age (continuous), ethnicity (Han, Other), household registration (urban, rural), education level (junior college and below, undergraduate, postgraduate and above), marriage status (unmarried, married), occupation (public institutions, private enterprise, student, other), and annual income (CNY; ≤100,000, 100,000–200,000, ≥200,000).

Pregnant status and histories—Participants were asked to report their number of pregnancies, gestational weeks, single or multiple birth, whether their Non-invasive Prenatal Testing was low-risk (no, yes), systematic ultrasonic examination was normal (no, yes); if they had history of multiple spontaneous abortions (no, yes), gestational hypertension, preeclampsia (no, yes), gestational diabetes (no, yes), giving birth to a fetus with developmental abnormalities (no, yes), low birth weight babies (no, yes), experiences of difficult labor, neonatal asphyxia rescue, and neonatal neurological damage (no, yes), having two or more preterm births (no, yes), immune diseases (no, yes), thyroid cancer, breast cancer, and other malignant tumors (no, yes), multiple and/or severe allergies to other vaccines or foods, such as eggs/seafood, or a history of severe anaphylactic reactions (no, yes), immunodeficiency diseases or severe hereditary immunodeficiency diseases (no, yes), major diseases in the mental, cardiovascular, respiratory, digestive, endocrine, hematological, and urogenital systems (no, yes), uncontrolled epilepsy and other progressive neurological diseases (no, yes).

Knowledge of influenza vaccine—2 questions, including “Have you ever heard of seasonal influenza vaccine?” (no, yes) and “Have you ever heard Influenza vaccines can effectively prevent respiratory infections and severe complications in pregnant women and their fetuses?” (no, yes).

Attitudes toward the seasonal influenza vaccine—Questions were asked as to whether pregnant women can receive the flu vaccine (no, yes); whether willing to receive the quadrivalent flu vaccine? (no, yes), the main reason of unwilling is (Not very familiar with the knowledge related to the quadrivalent influenza vaccine; Concerned about the vaccine’s effectiveness; Concerned about the vaccine’s safety (adverse reactions); Worried about the vaccine shortage; The vaccine is expensive; Others.); “Do you prefer a domestic or imported Seasonal influenza vaccine?” (indifferent, domestic, imported); and the price participants could afford to spend on an seasonal influenza vaccine (CNY; ≤200, 200–500, ≥500); “Which of the following situations is most likely to encourage you to get vaccinated” (multiple choices allowed) (Strong recommendation from a doctor; Vaccine included in the national immunization program; Strong recommendation from parents or family members; You or a family member has a history of flu-related illnesses; National health insurance covers the cost of vaccination; Most of your friends are willing to get vaccinated; Vaccine price drops to an acceptable range)?, and “If there is a clinical trial that allows you to receive a free vaccination with a quadrivalent influenza vaccine that has been proven safe for pregnant women and fetuses in animal studies, would you be willing to participate? (no, yes)”.

### 2.3. Statistical Analyses

For continuous variables following a normal distribution, values were reported as mean ± standard deviation (SD). Categorical variables were summarized using frequencies (n) and percentages (%). Between-group comparisons were conducted using two independent samples *t*-tests, while distribution differences in categorical variables were evaluated via the chi-square test. A univariate analysis was conducted to screen for potential variables (*p* < 0.05) that might be associated with the pregnant women’s willingness to vaccinate against influenza. Subsequently, a multivariate binary logistic regression model was constructed to further elucidate the independent effects of these variables on pregnant women’s vaccination willingness. The AMOS 23.0 software was used to create a mediation effect model to analyze the mediating effect of factors towards vaccination willingness, the Bootstrap method was used to test the existence of the mediating effect. All statistical analyses were performed with R4.2.1. All tests were conducted using two-sided tests, with *p* < 0.05 indicating statistical significance.

## 3. Results

### 3.1. Participants

In total, 1240 participants answered the questionnaire; 9 of them were excluded (5 questionnaires with logical errors and 4 with missing items over 95%), and a final 1231 participants were enrolled in this study. The average survey completion time was 5.1 min.

**Figure 1:**
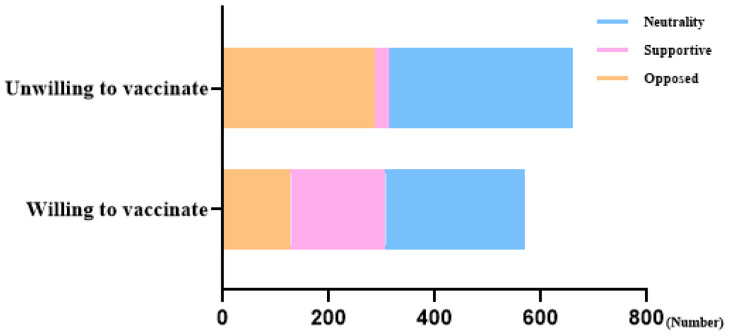
Willingness towards influenza vaccination for pregnant women.

Participants were categorized into either the willing or unwilling group based on their willingness to receive the seasonal influenza vaccination. Out of 1231 participants, 571 indicated a willingness to be vaccinated (Table 1, Figure 1). The results were displayed in Table 1. The mean age of the participants was 30.0 ± 3.9 years, with 89.8% identifying as Han ethnicity, and the mean body mass index (BMI) was 24.2 ± 8.5. Educational level, occupation, annual income, gestational weeks, ever having heard of the flu vaccine, pregnant women’s knowledge about the influenza vaccine, attitudes towards influenza vaccination for pregnant women, and the acceptable price of the vaccine showed statistical differences between the willing and unwilling groups.

The educational level was significantly higher in the willing group compared to the unwilling group. Specifically, 58 participants (10.2%) in the willing group held a postgraduate degree or higher, whereas only 50 participants (7.6%) in the unwilling group reported the same educational attainment. Additionally, 310 participants (54.3%) in the willing group were employed in public institutions, and 22 (3.9%) had an annual income of CNY ≥ 200,000. Moreover, the willing group demonstrated higher awareness of the influenza vaccine compared to the non-intervention group, and pregnant women in this group exhibited a more positive attitude toward influenza vaccination.

### 3.2. Influencing Factors of Willingness to Receive Seasonal Influenza Vaccination

To explore potential determinants of seasonal influenza vaccine acceptance among pregnant women, a multivariate logistic regression model was constructed (Table 2). Nine variables that demonstrated statistical significance in univariate screening were selected as independent predictors for inclusion in the final model. The analysis identified five key factors significantly associated with pregnant women’s willingness to receive seasonal influenza vaccination.

When comparing the willingness of pregnant women to receive the influenza vaccine based on annual income, those earning CNY ≥ 200,000 demonstrated significantly higher willingness to receive the influenza vaccine compared to those with an annual income of CNY ≤ 100,000 (Odds Ratio [OR]: 2.65, 95% Confidence Interval [CI]: 1.02–6.85). Pregnant women with a history of delivery difficulties exhibited only 10% of the willingness to vaccinate compared to those without such a history (OR: 0.10, 95% CI: 0.02–0.62). Additionally, pregnant women who possessed greater knowledge about the influenza vaccine were more likely to express a higher willingness to receive it (OR: 1.69, 95% CI: 1.28–2.23). Respondents who held supportive attitudes towards influenza vaccination for pregnant women showed a substantially higher willingness to get vaccinated (OR: 13.35, 95% CI: 8.26–21.55). Moreover, participants were more accepting of the vaccine if its price exceeded 500 yuan (OR: 2.29, 95% CI: 1.23–4.27).

### 3.3. Mediation Analysis

**Figure 2:**
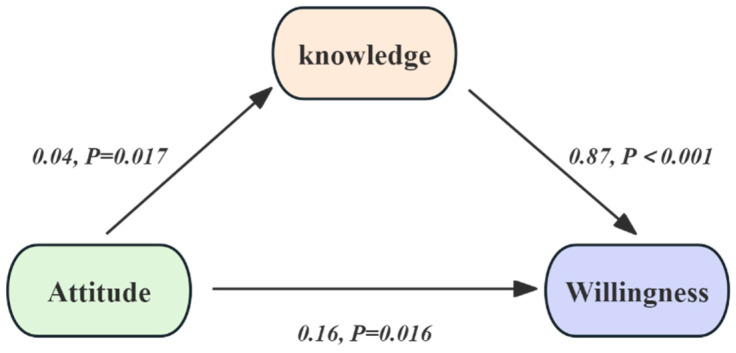
Attitudes towards influenza vaccination for pregnant women.

Model 1–3 exhibited the association between attitude (X) and willingness (Y) to receive the seasonal influenza vaccination was examined, with price (M1), history of dystocia (M2), and knowledge of the influenza vaccine (M3) considered as potential mediators. Notably, no mediation effects were observed through price or history of delivery difficulties. However, Model 3 revealed a significant partial mediation effect, as detailed in Table 3 and Figure 2. Specifically, a positive attitude toward influenza vaccine was found to significantly mediate willingness to receive the vaccination through knowledge of the influenza vaccine (β = 0.03, 95% CI: 0.01–0.06; *p* = 0.025), and the model fits well. This mediation resulted in a 3% increase in the likelihood of willingness, accounting for 16% of the total effect (Table 3).

## 4. Discussion

This research provides significant perspectives on the acceptance of seasonal influenza vaccination among pregnant women in China during pregnancy. The study results showed that annual income, history of delivery difficulties, knowledge about the influenza vaccine, attitudes towards influenza vaccination for pregnant women, and the acceptable price of the vaccine influence pregnant women’s willingness to be vaccinated. Further analysis revealed that a positive attitude toward the influenza vaccine enhances vaccination willingness, with improved knowledge about the vaccine acting as a critical mediating factor in this process.

The findings indicated that 53.6% of pregnant women demonstrated hesitancy toward receiving the influenza vaccine, primarily attributed to safety concerns and the prevalent belief that pharmaceutical interventions (including vaccinations) should be strictly avoided during pregnancy. This safety-related apprehension among Chinese pregnant women aligns with similar observations reported in international studies, including populations from Switzerland, Turkey, Canada, Scotland, Poland, the United States, Australia, Germany, and India [14,15,16,17,18,19,20,21]. In addition to the high perceived barriers associated with safety concerns, our participants also perceived minimal benefits from the influenza vaccine, likely due to a lack of information about its effectiveness. As a result, a substantial proportion of pregnant women in China adopted non-pharmaceutical interventions (NPIs) as preventive measures against influenza, including avoiding crowded environments and maintaining regular physical activity—strategies endorsed by obstetricians as effective for health preservation and influenza prevention. While NPIs demonstrate notable efficacy in reducing influenza transmission, evidence from past pandemics and extensive research consistently highlights vaccination and prophylactic antiviral therapy for exposed individuals as the most effective strategies for influenza prevention [7,22,23].

From a global perspective, the primary reasons for vaccine hesitancy stem from trust issues, risk perception, accessibility, socio-cultural factors, as well as trust in policies and governance. Among high-risk populations in South America, low trust is similarly a major driving factor [24]. A systematic review in Brazil found that information asymmetry, misinformation (or fake news), and inconvenient access to reliable information are key factors contributing to vaccine hesitancy [25]. European studies indicate that lower educational attainment and lower income levels are associated with higher rates of vaccine hesitancy [26].

In a sample of pregnant women in Turkey, exposure to negative information on social media increased the risk of vaccine hesitancy by 7–8 times. Global research shows pregnant women face diverse health info sources, from official to social media. Official channels may be inaccessible or untrusted, causing knowledge gaps. Social media, while convenient, spreads misinformation, creating fear and reducing vaccine acceptance, echoing prior findings [27,28]. Our findings indicate that a significant proportion of the pregnant women who participated in this study, particularly those in the unwilling group, were unaware of the benefits of the seasonal influenza vaccine. Through mediation analysis, we have uncovered that pregnant women’s attitudes toward the influenza vaccine are pivotal. These attitudes not only shape their willingness to get vaccinated but also exert an indirect influence via their cognition of the vaccine. From the Health Belief Model, positive attitudes suggest pregnant women perceive significant health benefits from vaccination and a clear threat from influenza, which in turn bolsters their vaccination willingness. This lack of awareness may stem from an insufficient understanding of the risks associated with severe influenza infection and its complications during pregnancy. Significantly, comparable patterns have been documented in other nations, including Australia and the United States, even though empirical data indicate that pregnant women experienced heightened vulnerability to severe pneumonia and respiratory failure during the 2009 influenza A/H1N1 pandemic [11,19]. Given that perceived threat, comprising both risk perception and susceptibility assessment, constitutes a pivotal determinant of health behavior adoption, initiatives such as enhancing awareness among pregnant women, obstetricians, and the broader population regarding the maternal-fetal risks of influenza infection represent a crucial initial step toward improving influenza vaccine coverage in China [12]. Economic constraints have been consistently identified as a major obstacle to health behavior compliance, with financial barriers demonstrating a negative correlation with vaccine acceptance [20,29]. In China, the quadrivalent influenza vaccine currently costs between CNY 100 and 350, imposing a considerable economic burden, particularly for low-income populations.

Efforts to encourage healthcare workers (HCWs) to provide pregnant women with information about the safety and effectiveness of the influenza vaccine may help dispel existing misconceptions. As numerous studies have demonstrated, HCWs play a pivotal role in promoting influenza vaccine uptake among pregnant women, and their recommendations serve as a critical “cue to action” for patients [30,31]. In particular, recommendations from obstetricians can significantly influence pregnant women’s acceptance of the seasonal influenza vaccine. However, studies conducted in various other countries have indicated that concerns about vaccine safety are a major barrier preventing obstetricians from recommending the influenza vaccine to pregnant women [32,33,34]. These anxieties arise not solely from concerns that immunization may trigger adverse maternal-fetal outcomes but also from fears that healthcare providers could face legal or professional repercussions for any associated complications.

Although China CDC has formulated technical protocols prioritizing pregnant women for influenza immunization, these recommendations have demonstrated limited penetration among obstetric practitioners [35]. The implementation of national-level policies or evidence-based guidelines, when systematically disseminated to healthcare providers, may function as institutional validation of vaccine safety. Such validation could mitigate clinical apprehensions regarding liability for adverse events, thereby enhancing obstetricians’ propensity to endorse vaccination. Both obstetric professionals and expectant mothers have emphasized the necessity for region-specific epidemiological evidence, with evidence suggesting that localized data could significantly improve immunization rates among high-risk populations in China. However, the current dearth of contextualized research findings presents a critical barrier, particularly considering the persistently low vaccination coverage among pregnant women. Sustained public health initiatives, leveraging trusted communication channels such as maternal-fetal medicine clinics and CDC platforms, are essential for fostering societal acceptance of influenza immunization across pregnant women, healthcare workers, and the broader community.

## 5. Limitations

This study exhibits several limitations that warrant explicit acknowledgment. Firstly, as a cross-sectional design, the research is inherently susceptible to measurement biases associated with self-reported data collection. Secondly, the questionnaire link was distributed via the official WeChat account rather than through face-to-face interviews; the respondents’ willingness to receive the seasonal influenza vaccine may be higher than that of the general population. Moreover, the sample might be biased towards women with higher socioeconomic status and better health literacy, which could potentially restrict the generalizability of the study’s findings. Thirdly, this study did not collect data on participants’ prior influenza vaccination history, a factor that may significantly influence pregnancy-related vaccination willingness through habitual behaviors or risk perception biases, which should be addressed in future research.

## 6. Conclusions

This study elucidated the factors influencing pregnant women’s willingness toward the seasonal influenza vaccine. The findings indicate that despite the potential benefits of influenza vaccines for pregnant women and their fetuses, a significant proportion of pregnant women remain hesitant or opposed to vaccination. The successful execution of an integrated immunization strategy necessitates coordinated interventions at both governmental and community levels. This requires the development of multi-tiered implementation frameworks that align national policy directives with localized public health initiatives. Additionally, greater attention should be directed toward individuals with lower incomes, those who are unaware of the benefits of the seasonal influenza vaccine, and those who decline vaccination. Lastly, the integration of seasonal influenza immunization programs with targeted screening initiatives, complemented by evidence-based health promotion and education frameworks, represents a critical strategy for reducing seasonal influenza incidence and minimizing its adverse perinatal consequences over the forthcoming decades.

## Figures and Tables

**Table 1:** Basic characteristics.

Variable	Total (*n* = 1231)	Willing (*n* = 571)	Unwilling (*n* = 660)	*p*
Age	30.0 ± 3.9	30.7 ± 3.9	30.8 ± 4.3	0.531
Ethnicity				0.479
Han	1105 (89.8%)	510 (46.2%)	595 (53.8%)	
Tujia ethnic group	87 (7.1%)	45 (51.7%)	42 (48.3%)	
Others	39 (3.2%)	16 (41.0%)	23 (59.0%)	
BMI	24.2 ± 8.5	24.1 ± 8.4	24.4 ± 8.6	0.591
Registered residence				0.310
Urban	619 (50.3%)	323 (48.9%)	296 (51.8%)	
Rural	612 (49.7%)	337 (51.1%)	275 (48.2%)	
Educational level		571	660	0.003
Junior college and below	536 (43.5%)	226 (39.6%)	310 (47.0%)	
Undergraduate	587 (47.7%)	287 (50.3%)	300 (45.5%)	
Postgraduate and above	108 (8.8%)	58 (10.2%)	50 (7.6%)	
Marriage status		571	660	0.215
Married	22 (1.8%)	14 (2.5%)	8 (1.2%)	
Unmarried	1204 (97.8%)	554 (97%)	650 (98.5%)	
Divorced	5 (0.4%)	3 (0.5%)	2 (0.3%)	
Occupation		571	660	0.010
Public institutions	611 (49.6%)	310 (54.3%)	301 (45.6%)	
Enterprise or private enterprise	617 (50.1%)	259 (45.4%)	358 (54.2%)	
Student	3 (0.2%)	2 (0.4%)	1 (0.2%)	
Annual income		571	660	0.013
CNY ≤ 100,000	824 (66.9%)	372 (65.1%)	452 (68.5%)	
CNY 100,000–200,000	378 (30.7%)	172 (30.1%)	196 (29.7%)	
CNY ≥ 200,000	29 (2.4%)	22 (3.9%)	7 (1.1%)	
Number of pregnancies				0.077
1	787 (63.9%)	382 (66.9%)	405 (61.4%)	
2	367 (46.6%)	123 (21.5%)	183 (27.7%)	
≥3	77 (21.0%)	66 (11.6%)	72 (10.9%)	
Gestational weeks	24.9 ± 8.7	25.5 ± 9.0	25.4 ± 8.6	0.043
Number of fetuses				0.325
Single fetus	1166 (94.7%)	537 (94.0%)	629 (95.3%)	
Multiple fetuses	65 (5.6%)	34 (6.3%)	31 (4.7%)	
Whether the prenatal checkup is normal				0.416
No	50 (4.1%)	26 (4.6%)	24 (3.6%)	
Yes	1181 (95.9%)	545 (95.4%)	636 (96.4%)	
History of spontaneous abortion				0.404
No	1085 (88.1%)	508 (89%)	577 (87.4%)	
Yes	146 (11.9%)	63 (11%)	83 (12.6%)	
History of gestational hypertension or preeclampsia				0.123
No	1216 (98.8%)	567 (99.3%)	649 (98.3%)	
Yes	15 (1.2%)	4 (0.7%)	11 (1.7%)	
History of gestational diabetes				0.959
No	1113 (90.4%)	516 (90.4%)	597 (90.5)	
Yes	118 (9.6%)	55 (9.6%)	63 (9.5%)	
Ever given birth to a abnormal fetus				0.512
No	1184 (96.2%)	547 (95.8%)	637 (96.5%)	
Yes	47 (3.8%)	24 (4.2%)	23 (3.5%)	
Ever given birth to a baby with low birth weight				0.744
No	1210 (98.3%)	562 (98.4%)	648 (98.2%)	
Yes	21 (1.7%)	9 (1.6%)	12 (1.8%)	
History of delivery difficulties				0.060
No	1220 (99.1%)	569 (99.6%)	651 (98.6%)	
Yes	11 (0.9%)	2 (0.4%)	9 (1.4%)	
History of premature birth				0.431
No	1222 (99.3%)	568 (99.5%)	654 (99.1%)	
Yes	9 (0.7%)	3 (0.5%)	6 (0.9%)	
History of immune diseases				0.769
No	1166 (94.7%)	542 (94.9%)	624 (94.5%)	
Yes	65 (5.3%)	29 (5.1%)	36 (5.5%)	
History of malignant tumors				0.362
No	1219 (99.0%)	567 (99.3%)	652 (98.8%)	
Yes	12 (1.0%)	4 (0.7%)	8 (1.2%)	
History of severe allergies				0.757
No	1213 (98.5%)	562 (98.4%)	651 (98.6%)	
Yes	18 (1.5%)	9 (1.6%)	9 (1.4%)	
History of immune deficiency diseases				0.818
No	1221 (98.2%)	566 (99.1%)	655 (99.2%)	
Yes	10 (0.8%)	5 (0.9%)	5 (0.8%)	
History of major systemic disease				0.177
No	1217 (98.9%)	562 (98.4%)	655 (99.2%)	
Yes	14 (1.1%)	9 (1.6%)	5 (0.8%)	
History of progressive neurological diseases				0.918
No	1229 (99.8%)	570 (99.8%)	659 (99.8%)	
Yes	2 (0.2%)	1 (0.2%)	1 (0.2%)	
Ever heard of the flu vaccine				0.004
No	241 (19.6%)	92 (16.1%)	149 (22.6%)	
Yes	990 (80.4%)	479 (83.9%)	511 (77.4%)	
Pregnant women’s knowledge about the influenza vaccine				0.000
No	740 (60.1%)	279 (48.9%)	461 (69.8%)	
Yes	491 (39.9%)	292 (51.1%)	199 (30.2%)	
Attitudes towards influenza vaccination for pregnant women				0.000
Opposed	414 (33.6%)	128 (22.4%)	286 (43.3%)	
Supportive	206 (16.7%)	179 (31.3%)	27 (4.1%)	
Neutrality	611 (49.6%)	264 (46.2%)	347 (52.6%)	
The acceptable price of the vaccine				0.000
CNY ≤ 200	768 (62.4%)	306 (53.6%)	462 (70.0%)	
CNY 200–500	413 (33.5%)	238 (41.7%)	175 (26.5%)	
CNY ≥ 500	50 (4.1%)	27 (4.7%)	23 (3.5%)	

**Table 2:** Factors influencing pregnant women’s willingness to receive seasonal influenza vaccination.

Variable	Univariate			Multivariate		
	β-Coefficient	OR (95%CI)	*p*	β-Coefficient	OR (95%CI)	*p*
Educational level			0.022			0.802
Junior college and below	Reference	1		Reference	1	
Undergraduate	0.27	1.31 (1.04–1.66)	0.024	0.02	1.11 (0.82–1.49)	0.510
Postgraduate and above	0.46	1.59 (1.05–2.41)	0.028	−0.04	1.10 (0.64–1.91)	0.726
Occupation			0.007			0.453
Public institutions	Reference	1		Reference	1	
Enterprise or private enterprise	−0.35	0.70 (0.56–0.88)	0.002	−0.22	0.87 (0.66–1.17)	0.358
Student	0.66	1.94 (0.18–21.5)	0.589	1.02	2.87 (0.24–34.50)	0.406
Annual income			0.009			0.015
CNY ≤ 100,000	Reference	1		Reference	1	
CNY 100,000–200,000	0.07	1.07 (0.84–1.37)	0.587	−0.28	0.76 (0.56–1.03)	0.076
CNY ≥ 200,000	1.34	3.82 (1.61–9.04)	0.002	0.97	2.65 (1.02–6.85)	0.045
Number of pregnancies			0.039			0.096
1	Reference	1		Reference	1	
2	−0.31	0.73 (0.57–0.94)	0.015	−0.27	0.76 (0.57–1.01)	0.062
≥3	0.08	1.09 (0.68–1.74)	0.724	0.20	1.22 (0.72–2.06)	0.454
Gestational weeks	0.00	1.00 (0.99–1.02)	0.799			
History of delivery difficulties						
No	Reference	1		Reference	1	
Yes	−1.37	0.25 (0.06–1.18)	0.081	−2.28	0.10 (0.02–0.62)	0.013
Ever heard of the flu vaccine						
No	Reference	1		Reference	1	
Yes	0.42	1.52 (1.14–2.03)	0.005	0.14	1.15 (0.81–1.63)	0.43
Pregnant women’s knowledge about the influenza vaccine						
No	Reference	1		Reference	1	
Yes	0.89	2.43 (1.92–3.06)	0.000	0.52	1.69 (1.28–2.23)	0.000
Attitudes towards influenza vaccination for pregnant women			0.000			0.000
Opposed	Reference	1		Reference	1	
Supportive	2.70	14.81 (9.40–23.35)	0.000	2.59	13.35 (8.26–21.55)	0.000
Neutrality	0.53	1.70 (1.31–2.21)	0.000	0.48	1.61 (1.22–2.12)	0.001
The acceptable price of the vaccine			0.000			
CNY ≤ 200	Reference	1		Reference	1	
CNY 200–500	0.72	2.05 (1.61–2.62)	0.000	0.76	2.15 (1.64–2.82)	0.000
CNY ≥ 500	0.57	1.77 (1.00–3.15)	0.051	0.83	2.29 (1.23–4.27)	0.009

Note: Univariate analysis identified variables associated with pregnant women’s influenza vaccination willingness, variables with statistical significance (*p* < 0.05) were subsequently included in a multivariate binary logistic regression model to assess their independent effects.

**Table 3:** Total, direct and indirect association of between attitude and willingness mediated via price, history of delivery difficulties and knowledge of the influenza vaccine.

Model	Total Association		Direct Association		Indirect Association		
	β (95% CI)	*p*	β (95% CI)	*p*	β (95% CI)	*p*	Proportion Mediated
Model 1: mediated via price							
Model 1	0.19 (0.08–0.30)	0.005	0.19 (0.09–0.30)	0.003	−0.01 (−0.03–0.01)	0.520	NA
Model 3: mediated via history of delivery difficulties							
Model 2	0.18 (0.08–0.30)	0.006	0.19 (0.09–0.30)	0.003	−0.01 (−0.05–0.000)	0.702	NA
Model 3: mediated via knowledge of the influenza vaccine							
Model 3	0.19 (0.08–0.30)	0.005	0.16 (0.05–0.27)	0.016	0.03 (0.01–0.06)	0.025	15.8%

## Data Availability

The data sets generated during and/or analyzed during this study are available from the corresponding author on reasonable request.
